# Marginal adaptation, physicochemical and rheological properties of treated dentin matrix hydrogel as a novel injectable pulp capping material for dentin regeneration

**DOI:** 10.1186/s12903-023-03677-6

**Published:** 2023-11-28

**Authors:** Ahmed A. Holiel, Eman M. Sedek

**Affiliations:** 1 Conservative Dentistry Department, Faculty of Dentistry, Alexandria University, Alexandria, Egypt; 2 Dental Biomaterials Department, Faculty of Dentistry, Alexandria University, Alexandria, Egypt

**Keywords:** Dentin matrix, Injectable scaffold, Pulp capping hydrogel, Marginal adaptation, Physiochemical properties, Rheology

## Abstract

**Background:**

Treated dentin matrix hydrogel (TDMH) has been introduced as a novel injectable direct pulp capping material. In this regard, this study aimed to evaluate its marginal adaptation, physicochemical and rheological properties for the development of clinically feasible TDMH.

**Methods:**

TDMH was applied to the pulp floor of prepared Class I cavities (n = 5), marginal adaptation was assessed by SEM at 1000 X magnification to detect gap between dentin and filling material. Five syringes were filled with TDMH and placed between the compression plates of a universal testing machine to evaluate injectability and gelation time was also evaluated by test vial inverting method. The microstructures of lyophilized TDMH were observed by SEM. Moreover, TDMH discs (n = 5) were prepared and the water uptake (%) was determined based on the equilibrium swelling theory state of hydrogels. Its solubility was measured after one week by the ISO standard method. Rheological behaviours of TDMH (n = 5) were analysed with a rotational rheometer by computing their complex shear modulus G* and their associated storage modulus (G′) and loss modulus (G′’). Statistical analysis was performed using F test (ANOVA) with repeated measures and Post Hoc Test (*p* = 0.05).

**Results:**

TDMH presented an overall 92.20 ± 2.95% of continuous margins. It exhibited gelation during the first minute, and injectability mean was 66 ± 0.36%. TDMH showed a highly porous structure, and the pores were interconnected with an average diameter about 5.09 ± 3.17 μm. Swelling equilibrium gradually reached at 6 days up to 377%. The prepared hydrogels and maintained their shape after absorbing over three times their original weight of water. TDMH fulfilled the requirements of ISO 6876, demonstrating a weight loss of 1.98 ± 0.09% and linear viscoelastic behaviour with G` 479.2 ± 12.7 and G`` 230.8 ± 13.8.

**Conclusions:**

TDMH provided good marginal adaptation, appropriate physicochemical and viscoelastic properties support its use as a novel direct pulp capping material in future clinical applications.

## Background

Direct pulp capping is a procedure in which a biocompatible material is applied on a vital exposed pulp to act as a barrier, protecting the dental pulp complex and preserving its vitality, ultimately aiming to induce dentin bridge formation by pulp cells [1, 2]. Marginal adaptation is a crucial physical property of an effective direct pulp capping material, as it ensures resistance to microleakage across its entire thickness [3]. Consequently, a well-adapted direct pulp capping material can lead to an enhanced biological response from the pulp. In contrast, inadequate adhesion, or adaptation of direct pulp capping materials to dentin can result in gaps, allowing fluid ingress into dentinal tubules, bacterial penetration, and pulp inflammation ultimately leading to abscess formation, tissue necrosis and treatment failure [4–6]. Current strategies employing biomaterials for pulp capping and dentin regeneration have notable limitations [7]. Some key limitations are the inflammatory reactions induced by the synthetic capping materials that can cause failure and the permeability of the newly formed dentin-like bridge that might enable bacterial recontamination of the tissues [5]. Additionally, the biomaterials currently used for healing dentin‐pulp tissues lack specific temporal and spatial control over biologic signaling required for progenitor cells’ homing and differentiation to fully restore the structure and function of the tissue [7, 8]. Therefore, there is significant potential in developing new dental materials that incorporate proteins or other factors that can stimulate regenerative processes.

Tissue engineering with the triad of dental pulp progenitor/stem cells, morphogens, and scaffolds could offer a valuable alternative approach for performing direct pulp capping procedures [9]. An alternative concept proposed for regenerating the dentin-pulp complex involves using cell-free scaffolds that incorporate bioactive compounds capable of homing, stimulating, and promoting the differentiation of stem/progenitor cells residing in the tissue [9, 10]. Recently, a novel injectable scaffold termed Treated Dentin Matrix Hydrogel has emerged, which combines alginate hydrogel as the matrix phase with TDM powder. This innovative material has shown significant potential for dentin regeneration and conservation of pulp vitality in direct pulp capping procedure [11–13].

Hydrogel-based scaffolds represent a distinct group of three-dimensional (3D) polymeric networks with high water content characterized by their hydrophilicity, biocompatibility, customizable degradation patterns and mechanical properties, and the capacity to incorporate various bioactive molecules [14]. Additionally, hydrogels offer a significant level of flexibility and elasticity, resembling the cell extracellular matrix which is crucial for providing the needed support during tissue regeneration [15]. Consequently, these unique characteristics make them an alternative choice for dentin-pulp complex regeneration [16]. Several characterization methods are available to determine the performance and quality of injectable hydrogels, and they should follow International Organization for Standardization (ISO) norms or American Society for Testing Materials (ASTM) standards [17]. These characterization techniques can be categorized into physicochemical, structural/morphological, thermal, mechanical, and biological assessments [18, 19].

The physicochemical characteristics of crosslinked hydrogels significantly influence both the final properties of the materials and their biological suitability [17]. Gelation time and kinetics for the injection of prepared hydrogels hold particular significance, especially for in situ applications and subsequent crosslinking once injected [20]. Furthermore, the swelling capacity of injectable hydrogels is critically important since various agents can be incorporated into these hydrogels to provide controlled release characteristics, and thus, supply an added value to these biomaterials [21]. Scaffolds should also have high porosity and interconnected pores, to facilitate vascularization, nutrients, and waste diffusion, as well as cellular migration [22, 23]. Aqueous hydrogels display interesting characteristic features when they are subjected to external stress; they can exhibit characteristics resembling those of a physical gel or solid, or they can flow like a liquid under specific conditions. Consequently, it is crucial to accurately assess their rheological properties. The viscoelastic property of aqueous-based polymer solutions plays a major role in terms of maintaining the similar elastic properties of host tissue to emulate the actual microenvironment for enhanced healing ability. Thus, these polymers should be fabricated in such a way as to maintain the native tissue´s rheological properties [24].

Selection of hydrogel-based scaffolds used for dentin-pulp complex regeneration can be based on the findings obtained from gelation time, injectability, porosity, swelling degree, and the viscoelastic characterization of selected biomaterials. Moreover, marginal adaptation and solubility are the key properties assessed for direct pulp capping. Therefore, the current study is expected to develop a clinically feasible TDMH as an innovative injectable material for dentin-pulp complex regeneration.

## Methods

### Fabrication of human TDMH

Treated dentin matrix hydrogel was conducted as previously described by Holiel et al. [11–13]. The formed hydrogel was loaded into a single syringe (5 mm chamber) to eliminate any excess solution and achieve a consistent mass of injectable hydrogel [11–13].

### Marginal adaptation of TDMH

Five sound molars were extracted for prosthetic reasons from patients aged 15 to 25 years who provided informed consent to donate their teeth for research purposes. This process adhered to the principles outlined in the Declaration of Helsinki and received ethical approval from the Faculty of Dentistry at Alexandria University, Egypt. (International No: IORG0008839). The sample size was determined using 80% power analysis at 95% confidence interval, relying on pilot study conducted with G power version 3.1.9.7. The teeth were cleaned by removing the hard deposits using a curette and the soft tissues through immersion in 5.25% Sodium hypochlorite solution for 10 min. Class I cavities were prepared with a 4-mm depth, a 4-mm mesiodistal width, and a 5-mm bucco–lingual width. Preparations were made using a cylindrical flat-ended fine diamond bur (837 B 012; Wilofa Diamant, Germany). The dimensions were continuously verified using a digital micrometer [4].

Freshly mixed TDMH was injected over the pulpal floor in one increment (1 mm) by using a single syringe (5 mm chamber). Then, the cavities were lined with a layer of resin modified glass ionomer cement (SDI, Bauswater victoria, Australia). All remaining cavity walls were then bonded with the universal adhesive (Single Bond Universal; 3 M ESPE, Deutschland GmbH, Seefeld, Germany) and was light cured for 10 s. The restorative material (Filtek Z250; 3 M ESPE, St. Paul, MN) was then placed into the cavity in increments and each increment was light cured for 20 s. Teeth were exposed to 5,000 thermal cycles between 5 and 55 °C in distilled water, with 30 s dwell time in each bath. They were then perpendicularly sectioned across their centers in a bucco–lingual direction with a low-speed saw (Isomet 1000; Buehler Ltd., Lake Bluff, IL) that was cooled by water [4]. A stereomicroscope (SZ61, Olympus, Japan) was used to validate the continuous margin. Additionally, all samples were examined by SEM (JSM 5600LV, JEOL, Japan) at an accelerating voltage of 20 kV. Marginal adaptation of the total length of the pulpal floor was assessed at X1000 magnification to identify any gaps between the dentin surface and the filling material. The classification of marginal adaptation followed the criteria established by Aggarwal et al. [25] into; continuous non gapped margin (continuous interface between the filling material and dentin with less than 1 μm gap) and gapped margin (interface between the filling material and dentin with gaps more than 1 μm wide). The SEM microphotographs were transferred to a computer and the gap was quantified using Image J software (Image J v1.44; US National Institutes of Health, Bethesda, MD, USA) [4, 26]. The in-built ruler of SEM (LEO 400 software) was used to measure the total length of pulpal floor. Subsequently, the length of any non-continuous margin (marginal gap > 1 μm) was assessed. This value was subtracted from the total length, and a percentage of “continuous margin” (CM) was calculated and recorded for each sample (n = 5).

### Physicochemical characterization of TDMH

#### Injectability of TDMH

**Fig. 1:**
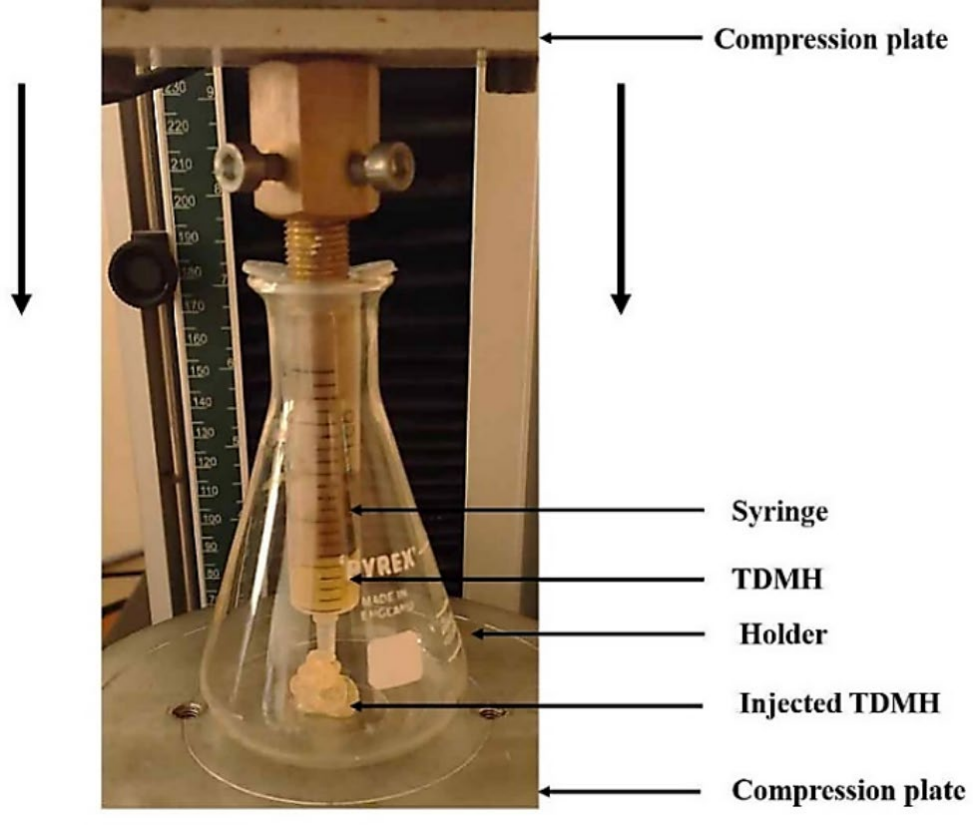
Showing the injectability of TDMH using Universal Testing Machine

The injectability of TDMH was evaluated by means of the experimental setup shown in Fig. 1. Five disposable syringes of 10 mL with a diameter of 10 mm were loaded with the prepared hydrogel and were positioned between the compression plates of a Universal Testing Machine. Then, the prepared hydrogel was extruded by applying a compression rate of 15 mm per minute up to a maximum force of 100 N. The injectability coefficient was calculated as the weight% of the hydrogel that was extruded from the syringe relative to the total mass of hydrogel initially placed in the syringe [17, 27].

#### Gelation time

**Fig. 2:**
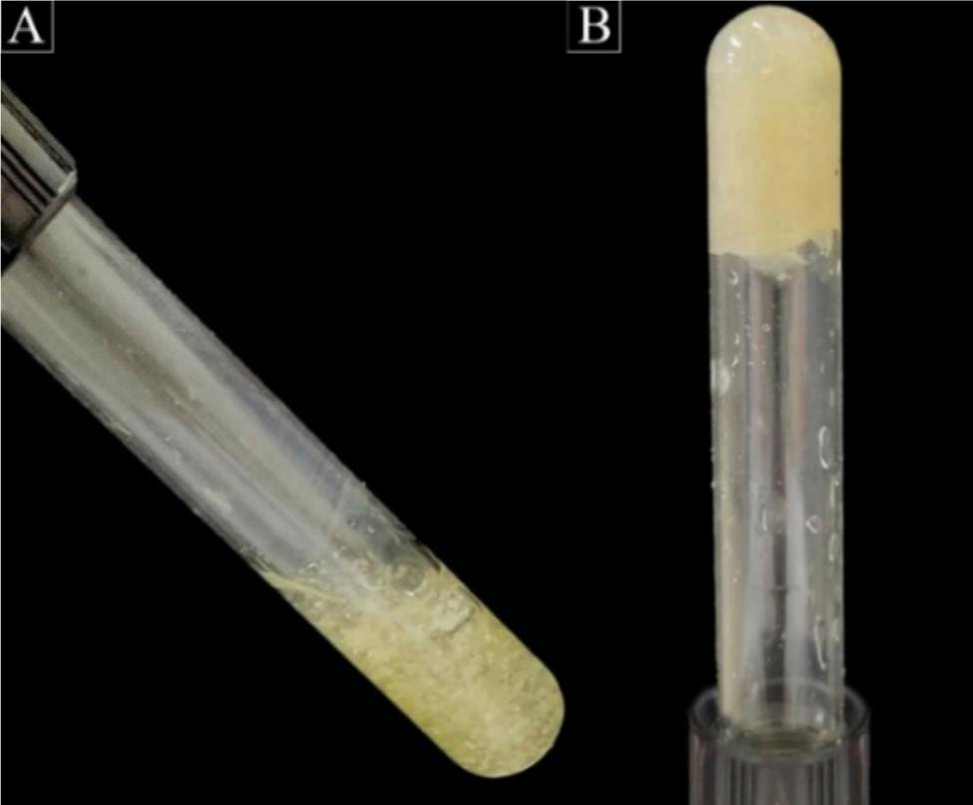
Example of a gelation time measurement by inverted vial method; (**A**) TDMH before crosslinking; (**B**) TDMH after crosslinking with CaCL_2_

Gelation time was defined as the time between the addition of cross-linking agents and the formation of the gel. The “tabletop” technique, test vial inverting method, which possesses a rheological basis was used to measure gelation (Fig. 2) [28, 29]. As described by Gupta et al. [30], when a test tube containing a solution is titled, it is defined as a sol phase if the solution deforms by flow, or a gel phase if there is no flow. The sol-gel transition time was determined by inverting the tube test horizontally every minute. The time at which the gel did not flow was recorded as the gelation time.

#### Morphological observations

The cross-sectional morphology of TDMH porous scaffolds was observed using SEM (JSM 5600LV, JEOL, Japan) at an accelerating voltage of 20 kV. After lyophilization, TDMH scaffolds were sputter-coated with gold prior to observation. Moreover, the diameters of the pores of the scaffolds were measured using Image J; and randomly selected for statistical analysis [31].

#### Determination of swelling degree of crosslinked TDMH

A total of 5 discs of dimensions 10 mm diameter × 8 mm height for TDMH were prepared and the swelling or water uptake (%) of crosslinked TDMH was calculated based on the equilibrium swelling theory state of hydrogels. The hydrogel was immersed in distilled water at pH 7.4 for 1–8 days and weighed at intervals (daily), with the hydrogel weight and volume increasing due to the swelling process. This step was repeated until no change in hydrogel weight was detected by using an electronic balance (RADWAG, AS 220.R2, Poland). This point is called the equilibrium swelling state of hydrogel, and then the equilibrium swollen hydrogel was freeze-dried. The water uptake (%) of hydrogels was given as follows [32]:

1 $$Water{\text{ }}uptake{\text{ }}\left( \% \right){\text{ }} = {\text{ }}[({W_s}-{W_0}){\text{ }}/{W_0}]{\text{ }} \times {\text{ }}100$$

Where Ws is the weight of the swollen hydrogel and W_0_ is the weight of the dried hydrogel.

#### Solubility evaluation

Five identical specimens were prepared using Split Teflon ring molds measuring 2 mm thick and 8 mm internal diameter. Each specimen was subjected to solubility testing according to the International Standards Organization (ISO) 6876 method [33] and with American Dental Association (ADA) specification No.30 [34]. The molds were placed on a glass plate covered with cellophane paper while the waxed thread was placed inside the mold that was filled to slight excess with prepared hydrogels. After filling the mold, another glass plate covered with cellophane paper was positioned on the top of mold applying gentle pressure to eliminate any excess hydrogel. Molds were stored in an incubator (Heraeus incubator, West Germany) at 37 °C and 95% humidity then the hydrogels were left to set [26].

The specimens were removed from the molds and the net weight of each specimen was recorded (W0) by high precision electrical weighting balance (RADWAG, AS 220.R2, Poland). Each specimen was suspended vertically in a clean glass beaker filled with distilled water at 37 °C in a special incubator for 7 days. At the end of the period, the specimens were removed from their glass beakers and rinsed with a little distilled water, blotted dry with absorbent paper, placed in a desiccator for 24 h, and then reweighed (W1) [26, 35]. Solubility percent for each sample was calculated as follows:

2 $$Wight{\text{ }}loss{\text{ }}\% {\text{ }} = {\text{ }}{W_0} - {W_1}/{W_0}{\text{ }} \times {\text{ }}100$$

### Rheological (viscoelastic) properties of TDMH

Hydrogels have typical characteristics of elastic and viscous nature, which are assessed experimentally through rheological measurements by computing their complex shear modulus G* and their associated storage modulus (G′) and loss modulus (G′’). G* (in Pa) describes the entire viscoelastic behavior of a sample. The storage modulus G’ for its part represents the elastic portion of the material while the loss modulus G′’ represent their viscous portion. Rheological behaviours of TDMH (n = 5) were analysed with a Rotational Rheometer (RS600, Thermo Hakke Co., Waltham, MA, USA). The specimens were then placed between two parallel circular plates (diameter of 20 mm) with a gap of 0.5 mm at 37 °C and 1 rad/s [24, 36].

### Statistical analysis of the data

All experiments were performed with sample groups of five repeats (*n* = 5). Categorical data were represented as numbers and percentages. All data are stated as mean ± standard deviation (SD). Statistical analysis was performed using IBM SPSS software package version 20.0. **(**Armonk, NY: IBM Corp**)**. ANOVA with repeated measures was used to compare between more than two periods and followed by Post Hoc test (adjusted Bonferroni) for pairwise comparison. Significance was accepted at *p* = 0.05.

## Results

### Marginal adaptation

**Fig. 3:**
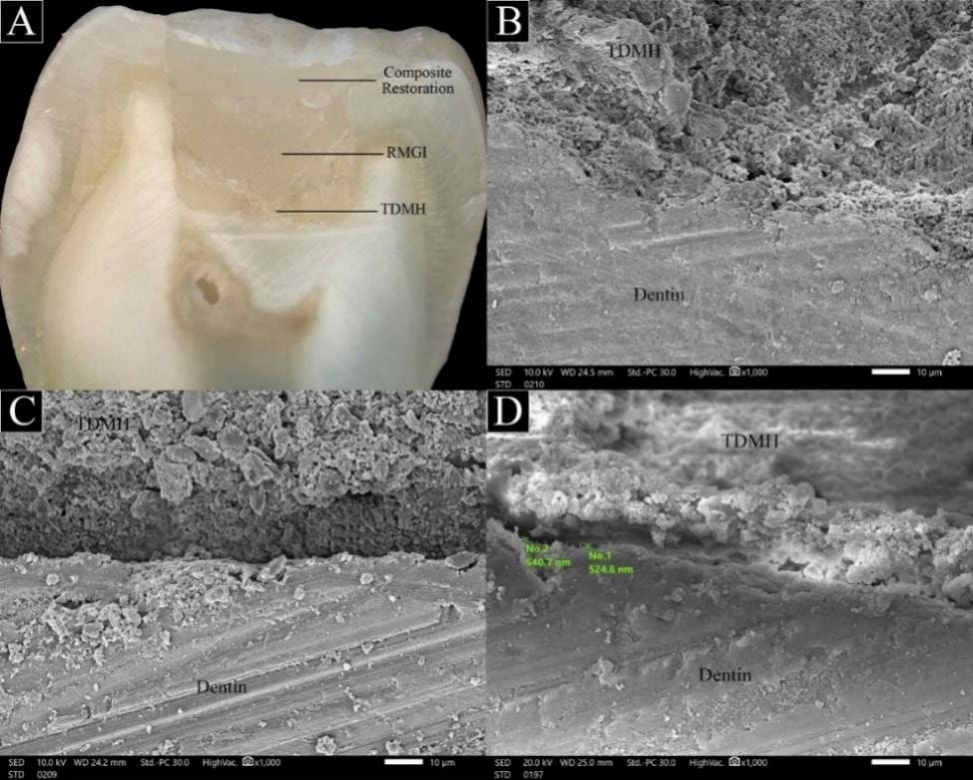
(**A**) stereomicroscope image showing TDMH in the pulp floor, RMGIC and composite filling material after thermocycling and sectioned in bucco–lingual direction. (**B**&**C**) Representative SEM images showing the marginal adaptation between TDMH and dentin with no deboned margins were observed at the interface in between; (**D**) SEM image showing gap formations through the interface (7.80%)

The marginal adaptation was recorded for treated dentin matrix hydrogel after sectioning the teeth across their centers in a bucco–lingual direction using a stereomicroscope and SEM. The percentage of continuous margin was about 92.20 ± 2.95 and non-continuous margin (gapped margin) about 7.80 ± 2.95. Representative stereomicroscope and SEM microphotographs are presented in Fig. 3 (A-D). Continuous margins were observed along the pulp floor for TDMH, showing no interfaces and good marginal adaptation to the dentinal wall. However, a gapped line appeared in some areas of the perimeter of the pulpal floor (ranging from 1 to 2.75 μm wide) according to SEM observations (Fig. 3D).

### Physicochemical characterization of TDMH

#### Gelation time and injectability

TDMH exhibited gelation during the first minute (1.0 ± 0.0), and their injectability mean was 66 ± 0.36%.

#### Morphological observation of crosslinked TDMH

**Fig. 4:**
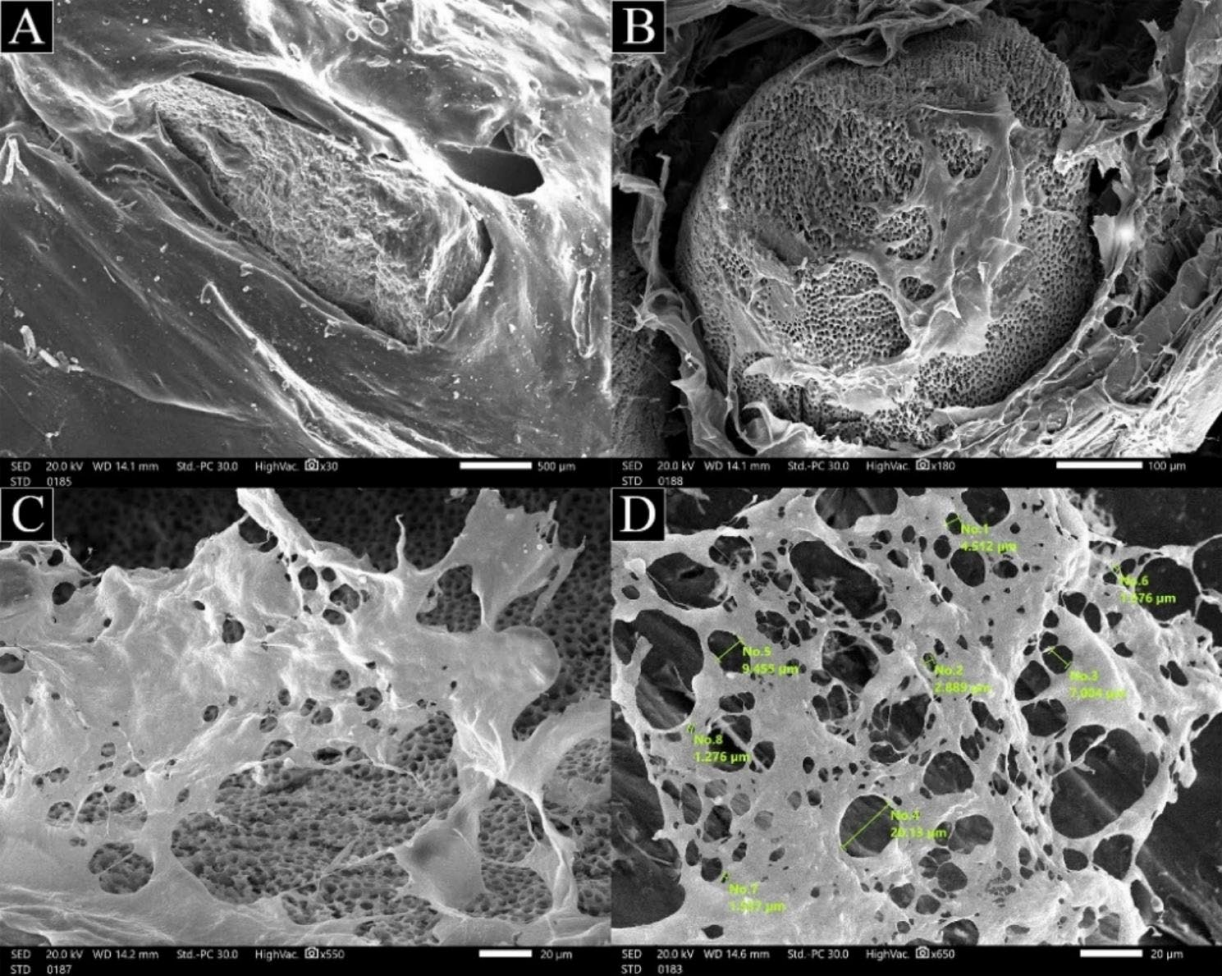
SEM images of the freeze-dried TDMH; (**A**) showed TDM particles impregnated within SA hydrogel; (**B**) SA hydrogel made a 3D network surrounding TDM and arrows showed dentinal tubules of TDM sufficiently exposed; (**C**) hydrogels exhibited a highly porous structure, and the pores were interconnected; (**D**) sizes of pores in TDMH were quantified

The microstructures of lyophilized TDMH were observed by SEM. The SEM images with a lower magnification were adopted to observe the distribution of TDM particles in the hydrogels (Fig. 4A). SA hydrogel made a 3D network surrounding TDM (Fig. 4B), the wall of the pore of TDMH was rough, and a lot of TDM particles were uniformly distributed in the wall surface. In a high magnification, all hydrogels exhibited a highly porous structure, and the pores were interconnected (Fig. 4C). Based on SEM images, the sizes of pores in TDMH were quantified (Fig. 4D). The average diameter of the pores of the prepared hydrogels was about 5.09 ± 3.17 μm.

#### Swelling degree of crosslinked TDMH

**Fig. 5:**
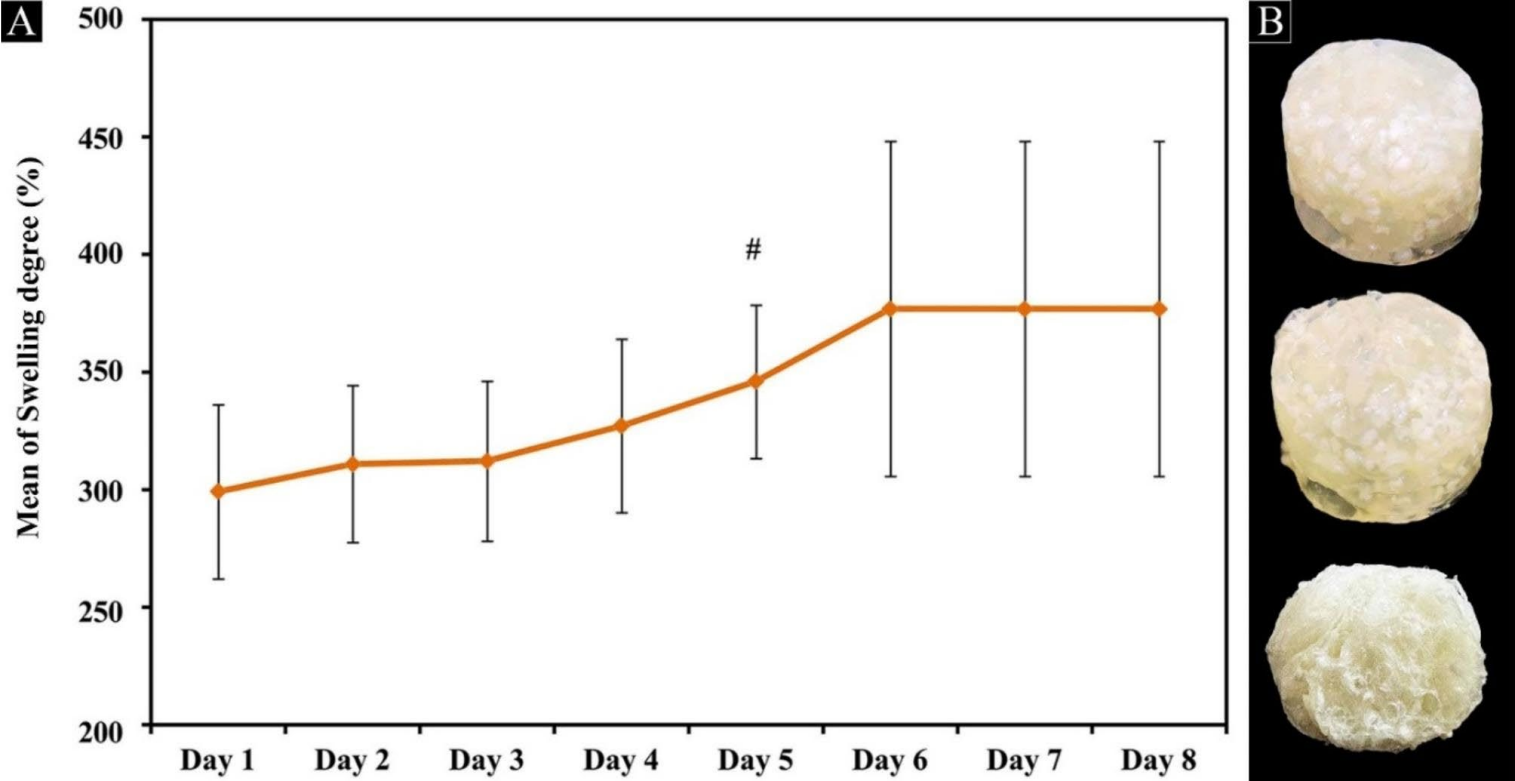
Swelling properties of TDMH. (**A**) Swelling kinetic curve of TDMH in PBS at 37℃. (**B**) The digital photographs of TDMH before, after equilibrium swelling at 6 days and after freeze drying

The swelling ratio was a quantified datum that was regarded as the capacity of the hydrogel absorbing liquid, and the result is shown in Fig. 5A. In the first 5 days, all samples exhibited a quick liquid absorption capacity, and the swelling ratios were about 299.3%, 311%, 312.3%, 327.2% and 346.1%, respectively. Then, the swelling ratio of all specimens expressed a slow upward trend. Finally, the swelling equilibrium gradually reached at 6 days, and this value was 377%. The photographs of TDMH immersed in PBS for 6 days are shown in Fig. 5B. The prepared hydrogels were not damaged and maintained their intact shape after absorbing over three times their original weight of water.

#### Solubility

The results of the solubility test (after 7 days) were analyzed. All the tested hydrogels fulfilled the requirements of ISO 6876 (solubility should not exceed 3%), demonstrating a weight loss of less than 3% with a mean and standard deviation of 1.98 ± 0.09.

### Rheological properties of TDMH

**Fig. 6:**
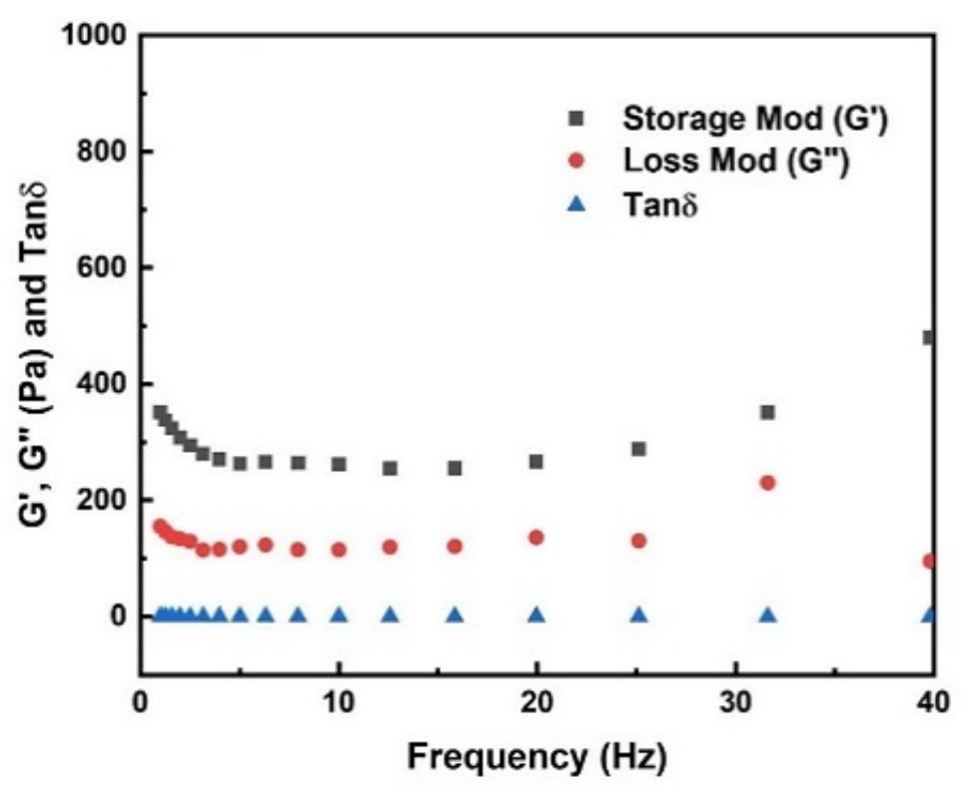
Linear viscoelastic material functions. Storage modulus, G`; loss modulus, G``; and tan *δ* versus strain amplitude behavior of TDMH at 10 rps and 37 °C

The dynamic material functions, namely, the storage modulus (G`), the loss modulus (G``), and tan *δ* (G``/G`) of the hydrogels, were measured over the strain magnitude range of 1–100% at 10 rps frequency. It was observed that the storage and loss moduli as well as tan *δ* values remained independent of the strain amplitude over the range of strain amplitudes studied, and, thus, in this strain amplitude range the hydrogels exhibited linear viscoelastic behavior. Furthermore, as shown in Fig. 6, the G′ was distinctly higher than the G′′ in the entire frequency range, the elastic behavior was dominantly relative to the viscous behavior. The means and standard deviations of the storage and loss moduli of prepared TDMH were 479.2 ± 12.7 and 230.8 ± 13.8 respectively.

## Discussion

For dentin–pulp complex regeneration, injectable hydrogels are considered a promising approach in the tissue engineering field. This is primarily attributed to their capacity to be easily injected into the tooth, seamlessly conforming to cavity defects. These hydrogels undergo in-situ gelation through the crosslinking of their precursors, ensuring effective and uniform filling of irregularities and defects within the tooth structure. [37]. The material should be in a flowable state during injection and solidify once injected, triggered by factors such as temperature, pH, light, enzymes, or the introduction of a crosslinking agent [23]. Additionally, the scaffold material should emulate natural biological tissues, acting as a 3 D matrix that not only supports cells but also replicates the extra cellular matrix. It should be capable of delivering bioactive molecules, have appropriate physicochemical and viscoelastic characteristics to optimize tissue regeneration [38, 39].

Our previous studies [11–13] extensively focused on the dentinogenic properties and biodegradation of the innovative injectable treated dentin matrix hydrogel, which is a combination of alginate hydrogel as the matrix component and TDM powder. Sodium alginate (SA) is frequently involved in the gelation process that is triggered by the presence of divalent cations, (usually Ca^2+^), forming structures known as hydrogels. The term hydrogel refers to a semisolid fluid where a polymer forms a three-dimensional network that swells in water [40]. Alginate hydrogels are soft in nature, mimicking most native tissues [41]. Moreover, alginate exhibits tunable mechanical characteristics that can be tailored to encompass a range of stiffnesses to match various tissues and presenting significant potential for cell delivery [42]. TDM was also confirmed to release dentinogenetic related proteins [43]. Consequently, TDMH induced a natural biological regeneration to reconstitute normal tissue continuum at the pulp-dentin border with degradation rate matched the rate of new dentin formation [11–13]. Additionally, TDMH demonstrated favorable biocompatibility over both short-term and extended periods with well-organized pulp tissue and almost no infiltration of inflammatory cells when used in direct pulp capping in human premolars [11]. However, to date, no study investigated marginal adaptation, physicochemical and rheological properties of TDMH as a novel injectable pulp capping material for dentin-pulp complex regeneration.

The three-dimensional hermetic seal is one of the most important requirements during direct pulp capping. When a material can effectively establish a biological seal, it promotes dentin bridge formation and regeneration [1, 4]. This seal involves several factors, including marginal adaptation, adhesion, solubility, and volume changes of the material used. Therefore, the gap size between dentin and the capping material serves as a measurable indicator of the material’s sealing ability [4, 44]. The evaluation of marginal adaptation of direct pulp capping materials by SEM can provide insights about their sealing ability [4]. The results of the present study showed that TDMH exhibited a good sealing ability that encourages its use as direct pulp capping material (gapped = 7.8% and non-gapped = 92.20%). Alazrag et al. [26] evaluated the marginal adaptation of mineral trioxide aggregate (MTA-Angelus) as direct pulp capping material that exhibited higher frequency distribution of gap presence (gapped = 50% and non-gapped = 50%). While Biodentine exhibited lower frequency distribution of gap presence (gapped = 25% and non-gapped = 75%). The good adaptation property of injectable TDMH may be attributed to the excellent rheological and viscoelastic properties of hydrogels that give them excellent shape adaptation and tissue adhesion, allowing them to be applied to irregularly shaped cavity defects for sustained and efficient repair function [45, 46]. Likewise, an insoluble barrier might be formed against microleakage.

Moreover, Injectability and gelation time are essential requirements for a biomaterial when it is performed in a minimally invasive manner as restoring dentin defect in direct pulp capping [23]. The technique for studying gelation is simple, reliable, and generally controlled by the mechanism responsible for the gel formation. While injectability is primarily associated with the rheological properties of the hydrogel, factors like concentration, viscosity, gelation process and gelation rate all impact the hydrogel’s injectability [47]. More crosslinking of alginate polymer can slow down the burst effect to an extent. Additionally, achieving an appropriate gelation rate is crucial; a rapid rate might impede the diffusion of the hydrogel polymer, while a slow rate could compromise the integrity of the hydrogel [23]. In the current study, TDMH exhibited gelation during the first minute, and their injectability mean was 66%. The gelation kinetics of the resulting TDMH could be controlled by varying the solubility of the aqueous CaCl_2_ crosslinking solution. In our study, CaCl_2_ solution with a 1:1 mass ratio to SA/TDM, along with glycerol, delayed the gelation process until the hydrogel was extruded from the syringe. Glycerol acted as a calcium sequestrant after its release, in addition to its plasticizing function. This dual function prevented premature cross-linking, which could have otherwise affected the injectability of the composites, ensuring the formulation of homogenous gels [48].

The hydrogel scaffolds used for dentin–pulp complex regeneration should have porous network structures. Pore size and interconnected porosity are important physicochemical properties that affect cell behavior and tissue formation [22, 23]. The diffusion of nutrients throughout the entire structure of the gel and removal of metabolic wastes are ensured by uniform pore size and distribution. Varied pore sizes are linked to distinct cellular behaviors [46]. The morphological characterization of TDMH in this study showed a porous structure with a moderate pore size (5.09 μm). These porous channels could serve as transport channels for vascularization, nutrients, and waste diffusion, as well as cellular migration, adhesion, and proliferation, which are biological events that are essential for dentin regeneration [23, 38]. Huang et al. [49] concluded that hydrogels with small pore sizes (2–50 nm) utilize substantial surface area for drug loading, leading to a favorable therapeutic outcome. While Schröter et al. [50] found hydrogels with a moderate pore size (5–10 μm) could enhance hydroxyapatite formation and facilitate the incorporation of bone morphogenetic proteins by accelerating the exchange of mineral ions such as calcium, magnesium, zinc and others. Large pore size (above 100 μm) was found to significantly accelerate cell migration and adhesiveness as concluded by Li Y et al. [51].

Another significant concern is enhancing swelling degree in hydrogel preparation for dentin-pulp complex regeneration. This is critical as it enables the controlled release of signaling molecules, along with calcium and phosphate, from the scaffold for tissue mineralization. It also provides the necessary features for improving surface morphology [32]. Hydrogels must possess the ability to absorb liquids to enrich the nutrients that can support cell growth and maintain a moist environment conducive to tissue regeneration. Hydrogels interact with aqueous solutions and swell to a certain equilibrium and retain a substantial amount of water within their structure [21, 52]. However, having a controllable swelling rate to allow sustained release is crucial. A slow swelling rate can delay the release of bioactive materials, typically due to a compact and less porous structure. While, a rapid and high swelling of the hydrogel, along with a porous morphology and weak crosslinking density, can lead to a burst release, which may have a negative impact on tissue formation [53]. The current study revealed that the swelling ratio of all TDMH specimens exhibited a slow upward trend, eventually reaching equilibrium after six days. Notably, TDMH remained structurally intact even after absorbing over three times their original weight of water. This could be attributed to TDM along with SA hydrogel could occupy more space than should be held by water, further giving rise to reduced rate of swelling. Furthermore, hydrogel, which relies on the Schiff base reaction, could absorb a substantial amount of liquid while retaining its own shape, rendering it suitable tissue engineering.

Furthermore, solubility of TDMH was tested in this study. Limited solubility is a favorable characteristic of direct pulp capping material for achieving a durable long-term seal, preventing microleakage [1, 4]. Solubility was evaluated by ISO 6876:2012 based on previous studies [54–56] evaluated the solubility of different pulp capping materials using ISO 6876 clause 4.3.6 after a period of 7 days as the most widely accepted time frame [26, 57]. In this study, all the tested hydrogels fulfilled the requirements of ISO standards, demonstrating a weight loss of 1.98 ± 0.09%. This could be attributed to the presence of calcium ions in TDMH as a crosslinker. These crosslinked ions stabilize alginate chains, forming a gel structure that facilitates effective crosslinking between polymer chains and limits solubility, with more freely movable chains that bind and entrap favorable quantities of biological fluids [41, 42]. In addition, the inclusion of TDM particles in the prepared scaffolds can result in reduced solubility of TDMH by lowering its swelling ratio, consequently leading to decreased solubility. Alazrag et al. [26] evaluated the solubility of MTA after 7 days with a mean value of 1.74 ± 0.4%. While Biodentine exhibited the highest solubility of 3.36 ± 0.3%. They attributed the lower solubility of MTA-Angelus than Biodentine to the absence of Calcium carbonate and presence of setting accelerator in its composition that result in shortening of the setting time [57].

The rheological properties of the hydrogel can be tailored by modifying factors such as polymer and cross-linker concentrations, polymer molecular weight, concentration of polymer reactive groups, ambient temperature and pH, preparation technique, and gelation method [58, 59]. Direct pulp capping materials should exhibit low viscoelastic properties to facilitate proper flow and adaptation within irregular dentin defects. This may help reduce the chance of gaps forming at the tooth–material interface, which could result in restorative leakage and treatment failure. On the other hand, a higher viscosity of the material could negatively impact its ease of handling, potentially complicating the filling procedures [60]. The findings of the rheological properties from this study indicate that the viscoelastic characteristics of TDMH resemble the behavior typically seen in microgels. The existence of gel-like behavior is indicated by the frequency independent behavior of the storage modulus (G`) and the loss modulus (G``) coupled with the G` and G`` versus the frequency data exhibiting parallel behavior [61]. For all samples, the G′ was distinctly higher than the G′′ in the entire frequency range, the elastic behavior was dominantly relative to the viscous behavior. This meant that the changes in frequency did not destroy the structure of hydrogels and that hydrogels maintained a solid state. In addition, the G′ remained stable across the whole frequency range, revealing the nature of covalent cross-linking hydrogels.

If the rheological properties of a hydrogel closely mimic those of the native pulp tissue, it can potentially offer needed support for dental pulp stem cells to undergo differentiation into odontoblast-like cells and promote dentin tissue formation in direct pulp capping procedure [16]. The native dental pulp tissue consists of collagen fibers, proteoglycans, and dental pulp cells together with water and electrolytes [16]. The proteoglycans form interconnected networks that can store deformational energy, while cross-linked fibrous collagens provide tensile stiffness and strength [24]. The viscoelastic properties of connective tissue are influenced by the existence of macromolecules like collagens and proteoglycans, along with their interactions with cells and other components of ECM. Additionally, these properties are also influenced by the hydrodynamics of the interstitial fluid flow [61].

The current study revealed that TDMH ranged within the described native pulp tissue storage G` and loss modulus G`` values (around 100 Pa and 10 Pa, respectively) [62], validating this novel injectable scaffold as 3D matrix able to mimic the rheological properties of native pulp tissues that encouraged odontogenic differentiation and new dentin tissue formation. Erisken C et al. [62] found that storage and loss moduli of 2% alginate and 2% agarose were significantly higher than those of than native pulp tissues (in the ranges of 1000–10,000 and 300–1,000, respectively). In addition, oxidized alginate-hybrid-hydroxyapatite nanoparticles showed a G` of 390 Pa and the G′ of the hydrogels increased with increasing the concentration of hydroxyapatite nanoparticles [62]. Alternatively, it should be noted that the difference in viscoelastic properties of TDMH from the native pulp tissue can be minimized by altering the gelation agent concentration as well as the degree of cross-linking. In this regard, the viscoelastic behavior is regulated by the density of entanglements among macromolecules, which, in turn, is influenced by the distribution of molecular weights within the polymer.

## Conclusions

This study emphasizes that TDMH provided good marginal adaptation, appropriate physicochemical and viscoelastic properties which support its use as a novel direct pulp capping material in future clinical applications. Further studies to construct TDMH as a three-dimensional bio-printing are recommended for individual customization in clinical application to obtain the best morphological match and achieve an individualized regenerative goal.

## Data Availability

The datasets used and analysed during the current study are available from the corresponding author on reasonable request.
